# CELLO2GO: A Web Server for Protein subCELlular LOcalization Prediction with Functional Gene Ontology Annotation

**DOI:** 10.1371/journal.pone.0099368

**Published:** 2014-06-09

**Authors:** Chin-Sheng Yu, Chih-Wen Cheng, Wen-Chi Su, Kuei-Chung Chang, Shao-Wei Huang, Jenn-Kang Hwang, Chih-Hao Lu

**Affiliations:** 1 Department of Information Engineering and Computer Science, Feng Chia University, Taichung, Taiwan; 2 Master's Program in Biomedical Informatics and Biomedical Engineering, Feng Chia University, Taichung, Taiwan; 3 Department of Medical Informatics, Tzu Chi University, Hualien, Taiwan; 4 Institute of Bioinformatics and Systems Biology, National Chiao Tung University, Hsinchu, Taiwan; 5 Center of Bioinformatics Research, National Chiao Tung University, Hsinchu, Taiwan; 6 Graduate Institute of Basic Medical Science, China Medical University, Taichung, Taiwan; CSIR-Institute of Microbial Technology, India

## Abstract

CELLO2GO (http://cello.life.nctu.edu.tw/cello2go/) is a publicly available, web-based system for screening various properties of a targeted protein and its subcellular localization. Herein, we describe how this platform is used to obtain a brief or detailed gene ontology (GO)-type categories, including subcellular localization(s), for the queried proteins by combining the CELLO localization-predicting and BLAST homology-searching approaches. Given a query protein sequence, CELLO2GO uses BLAST to search for homologous sequences that are GO annotated in an in-house database derived from the UniProt KnowledgeBase database. At the same time, CELLO attempts predict at least one subcellular localization on the basis of the species in which the protein is found. When homologs for the query sequence have been identified, the number of terms found for each of their GO categories, i.e., cellular compartment, molecular function, and biological process, are summed and presented as pie charts representing possible functional annotations for the queried protein. Although the experimental subcellular localization of a protein may not be known, and thus not annotated, CELLO can confidentially suggest a subcellular localization. CELLO2GO should be a useful tool for research involving complex subcellular systems because it combines CELLO and BLAST into one platform and its output is easily manipulated such that the user-specific questions may be readily addressed.

## Introduction

It is generally believed that the function of a protein is related to its subcellular localization, because the environment of a protein provides part of the relevant context necessary for function. However, even if a subcellular localization is known, it should not be the only piece of acquired evidence as additional information concerning the protein should be helpful during the course of a biological study related to the protein. To obtain a global overview of the function(s) that an uncharacterized protein might have *in vivo*, the Gene Ontology (GO) annotations, e.g., cellular location, molecular function, and biological process, of homologous proteins [1] are often useful. To rapidly and accurately find the appropriate GO annotations and determine the possible relationships within a given set of proteins, BLAST [2] is often used to search for proteins with similar sequences and known functions [3] so that functional GO-category annotations can be made [4], [5]. But when a BLAST search is not productive, advanced computational tools are often used to provide clues that will enable prediction of GO-like terms. Therefore, many programs have been developed to predict the function [6] and the subcellular localization of a targeted protein. [7], [8], [9], [10], [11], [12], [13], [14], [15], [16], [17], [18], [19], [20], [21], [22], [23] Some of these programs provide additional information, e.g., protein-protein interactions [14], [19] or three-dimensional structure comparisons [18], although most just attempt to determine the subcellular compartment of the targeted protein. Additionally, studies have found that the more similar protein sequences are, the greater the likelihood that proteins with similar sequences will be found in the same subcellular localization [17], [22]. A hybrid approach combining machine learning and homology searching also can provide accurate subcellular-localization predictions. [22] The reason why certain computational tools provide improved subcellular localization prediction appears to be that GO information [8], [9], [10], [12], [15], [20], [21] or a homology-based modular structure comparison [23] is included in the prediction routine. However, if homologs for the protein of interest are not GO annotated or if a signature(s) and sequences similar to that of the query protein are not found in a relevant, searched database, such as InterPro [24], then a prediction cannot made. Among the programs that do not use a homology-based approach, CELLO [22], [25] performs as well as one that requires a much larger amount of training data [26]. CELLO is easy to use and has a fast computational time as has been noted [27], [28]. The ability of CELLO to identify possible subcellular localizations for targeted proteins is especially important for proteomic research when the compartments are of special interest, but when homologs have not been found by BLAST or when GO annotations are few in number.

Notably, a web service that conveniently provides comprehensive functional and localization annotation, and can correlate the two has not been available. By extending the hybrid approach [22], we report herein the implementation of the CELLO2GO server (http://cello.life.nctu.edu.tw/cello2go/), which provides brief and/or detailed annotations of GO terms related to homologs of a query protein found by BLAST searching in combination with a CELLO-predicted subcellular localization(s) for the queried protein. In addition, CELLO2GO can be used to identify protein sequences and their associated GO and CELLO terms when query sequences are submitted in batch mode. We describe how BLAST in CELLO2GO collects and displays the available GO-based annotations of homologous sequences found in in-house database derived from the UniProt KnowledgeBase for a query protein or a set of query proteins from a wide variety of organisms [29], while, at the same time, CELLO in CELLO2GO performs the same tasks for subcellular localization(s). CELLO2GO output is presented as GOOGLE-created pie graphs and hyperlinks, which clearly display the evidence for each annotation. We believe that CELLO2GO will be of assistance in future genomic and proteomic research because it is easy to use, and its results can be manipulated to provide information specific to the concerns of the user.

## Methods

**Figure 1:**
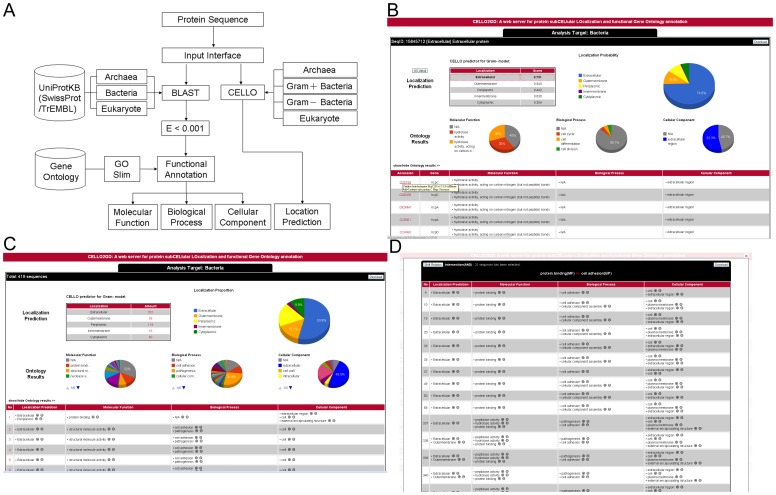
Flowchart for CELLO2GO and examples of the input and output interfaces. (A) The flowchart for annotation of a protein sequence used by CELLO2GO. The search databases used in the work are modified forms of the UniProtKB/SwissProt and UniProtKB/TrEMBL databases. (B) The CELLO2GO output page for a multiple-sequence query, which provides four pie charts, one for the localization predictions returned by CELLO (upper right) and three for the GO terms returned by BLAST for each query sequence. The list, which can be hidden, below the pie charts presents the CELLO-predicted subcellular localizations and the associated GO annotations in the order that the sequences were submitted. (C) The CELLO2GO output page for a single sequence query, which provides four pie charts, one for the CELLO-predicted subcellular localizations (upper right) and three for the GO terms returned by BLAST for the retrieved homologous sequences. The list, which can be hidden, below the pie graphs presents the CELLO-predicted subcellular localization(s) and the associated GO annotations in the order that the homologous sequences were found by BLAST. (D) By clicking on the GO-term list in (B), a new list of submitted sequence entries with the same GO term is returned.

The flowchart for CELLO2GO is illustrated in Figure 1A. If the species from which the sequence is derived is known, BLAST will immediately search for homologs within the corresponding sub-database of an in-house database(s) (see below for information concerning the in-house databases); if not, the entire database(s) can be searched. By default, all GO terms for each retrieved homolog are collected form the database(s) and grouped into one three GO categories. The first in-house database to be searched is derived from the UniProtKB/SwissProt, which currently contains the best documented and most complete function-annotated sequences. The server can also search for GO annotations defined by InterPro if functional information is absent from the homolog records or for GO terms recorded in the UniProtKB/TrEMBL database if no homologs are found in InterPro and the UniProtKB/SwissProt-derived databases. Separately, CELLO attempts to predict a subcellular localization(s) for the query protein using its most recently trained model. CELLO may also be implemented after the organism type has been identified by BLAST searching, e.g., after identifying the query sequence as from a Gram-positive or Gram-negative bacterium. For each query sequence, the CELLO2GO results are displayed as Google-created pie charts at the output interface and represent how often a potential GO annotation has associated with all retrieved homologs and a possible localization it is in. The names of all retrieved protein and their functions presented in the pie charts are also listed on the output page. After clicking on an ontology term of interest in the list below the pie charts, the retrieved proteins in the searched database(s) having the same ontology are shown.

When sequences are batch inputted into CELLO2GO, the server processes the data in the same manner as when one sequence is inputted at a time. The annotations for each protein are retained while additional sequences are processed. After subjecting a set of proteins - e.g., from proteomic dataset – with various functions to CELLO2GO, the output GO annotations and CELLO-identified subcellular localizations of the inputted sequences are displayed as pie charts allowing the user to visualize how many GO annotations and subcellular localization are associated with the inputted sequence set. The name of the proteins associated with each corresponding sequence and its annotations to be calculated for the pie chart are listed in the same page, too. The inputted sequences in a set that share common ontology features can be grouped by selecting a single ontology term in the list one at a time. When the GO annotations of one sequence in a set of input sequences are of interest, by clicking on its number in the first column of the output list, its GO annotations are displayed in detail, the pie charts are recreated to reflect the GO annotations of only that sequence, and the list below the pie charts now reflects the sequences homologous to the input sequence of interest according to their BLAST-retrieved UniProtKB/SwissProt entry identifiers, gene names, and associated GO annotations, in the order of their E-values. Shown above the “Ontology Results” caption is a button labeled “GO detail” that, when clicked, allows the user to switch between detail GO and GO-slim terms.

### Background Databases

To focus the BLAST search on sequences from similar organisms and to accelerate data processing, we prepared, in April, 2013, a databases of all non-redundant proteins from the UniProtKB/SwissProt database that contained 539616 protein records (separated into 16316 viral, 18993 archaeal, 328774 bacterial, and 175533 eukaryotic sequences) and a database from the UniProtKB/TrEMBL database (32051161 protein records, separated into 1599881 viral, 428746 archaeal, 22935705 bacterial, and 7086829 eukaryotic sequences). We formatted and indexed these sequences so that the user needs to BLAST search only the appropriate sub-database when the species for the sequence(s) is known. All fundamental information for the in-housed databases was formatted as a MySQL database. In the single sequence mode, after the query sequence has been compared by BLAST with those in the user-selected sub-database, homologous sequences are returned if their E-value is the same as or smaller than a user-specified threshold (default E-value is 0.001), and at the same time the GO terms are retrieved automatically for the homologous entries, which is the most time-consuming step for a multiple input sequence submission.

For homologs, their GO terms are subdivided into molecular functions, biological processes, and cellular components and the number of terms found in each category is summed. GO terms are also summed as their simplified/generalized forms, the GO slims [30], for more robust or other specific problems.

### Generation of GO slims

Even through the UniProtKB/SwissProt database contains the most detailed information available in any database, the amount of information differs for each entry, and this difference in information content is reflected in tree-like GO constructions of the categories, i.e., the more data we have, the better developed the trees. When we would like to just scrutinize and obtain an overview of a GO hierarchy, the generic GO-slim categories (http://www.geneontology.org/GO.slims.shtml), which are not species specific, are suitable for this task. For the output, the GO slims were manipulated by tracing back to the ontological roots of the proteins using the GO terms in the UniProtKB/SwissProt database. For example, for a functional annotation of entry P27989, we can trace the path from the deepest GO term, “nickel cation binding” to its root by passing through the GO terms “transition-metal-ion binding,” “cation binding,” “ion binding,” and “binding.” In this case, only the GO term “ion binding” is retained and denoted as the GO slim term. CELLO2GO counts all traced GO slims as general GO annotations.

### Subcellular Localization prediction

To complement incomplete annotations in the background database, a homology-ontology annotation retrieved by BLAST should be accompanied by an accurate subcellular localization prediction for each homologous sequence. CELLO has been shown to be helpful for the prediction of subcellular localizations of the proteins found in a proteomic data. [28] Using multiple, integrated machine-learned classifiers, CELLO predicts which of four subcellular localizations in archaea and in Gram-positive bacteria, five subcellular localizations in Gram-negative bacteria, and twelve subcellular localizations in eukaryotes that the targeted protein might be found in, with the four archaeal and Gram-positive bacterial localizations being the extracellular space, the cell wall, the cytoplasmic membrane, and the cytoplasm; the five Gram-positive bacterial localizations being the extracellular space, the outer membrane, the periplasmic and cytoplasmic (inner) membranes, and the cytoplasm; and the 12 eukaryotic localizations being chloroplasts, the cytoplasm, the cytoskeleton, the endoplasmic reticulum, the extracellular/secretory space, the Golgi, lysosomes, mitochondria, the nucleus, peroxisomes, the plasma membrane, and vacuoles. Due to subcellular data increased exponentially over the years, CELLO has been trained on latest models and denoted as update version wrapping in CELLO2GO. And the resultant datasets used for prediction and evaluation is from PSORTb3.0 [23].

### Evaluation measure

CELLO2GO is not meant for prediction of a protein's function(s), but for correlating one protein with another through the same functional annotation. To achieve this goal, it is necessary to obtain as many functional annotations as possible. Retrieved GO annotations are retained for outputted sequences similar to that of the query protein. Even when dealing with multidomain proteins, BLAST, which uses a local alignment approach, can easily find all similar sequences in the database(s) with their functional annotations provided as output. It is very important to functionally annotate each protein in the output set even for those proteins that are multifunction and/or promiscuous, so that the CELLO output complements any incomplete GO cellular-component ontology annotations. For our purposes, we treated the CELLO2GO results for a given sequence in our example (see below) as correct if any collected GO-slim cellular-component annotation(s) was also correct.

To validate that CELLO2GO can correctly identify the subcellular localization of a query protein, we used the archaea, and the bacterial Gram-positive and Gram-negative benchmark datasets found in PSORTb3.0 [23], which we denoted PS30Arch, PS30GP, and PS30GN, respectively. We also used the newly documented Gram-negative *Pseudomonas aeruginosa* PA01 genome/proteome sequence dataset [31] (http://www.pseudomonas.com/), which contains, in part, hypothetical and uncharacterized proteins that can be difficult to functionally annotate because homologs or useful GO annotations would be are missing in the UniProtKB/SwissProt/TrEMBL databases.

We then ascertained if for a given protein its subcellular localization(s) found by CELLO and BLAST (defined as a GO slim(s) agreed. For example, if a protein was assigned the GO-slim terms “external encapsulating structure”, “extracellular region” or “extracellular space”, then the associated CELLO term would be “extracellular”. And the GO-slim term “plasma membrane” associated with CELLO terms “outer membrane” and “inner membrane”, the GO-slim terms “cell” and “intracellular” associated with CELLO term ”periplasmic”, and the GO-slim term “cytoplasm” associated with CELLO term “cytoplasmic”, respectively. Because CELLO2GO uses a hybrid procedure [22], CELLO2GO identifies potential subcellular localization of the query protein using the GO cellular**-**component annotation of homologous sequences retrieved by BLAST along with other GO annotations and/or the CELLO-predicted localization(s) if BLAST-retrieved sequences are not associated with a GO cellular component annotation or if homologs are not found. We calculated the prediction accuracy, *Q_i_,* which is defined as *Q_i_* = *c_i_*/*n_i_* ×100, to assess the performance of the CELLO prediction, where *c_i_* is the number of correct CELLO predictions for the localization *i* (e.g., one of the five Gram-negative bacterial localizations), and *n_i_* is the number of sequences. The overall accuracy is given by



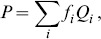
where *f_i_* = *n_i_*/*N*, and *N* is the total number of sequences.

### Web Server Description

The web pages for CELLO2GO are shown in Figure 1B-D. Starting at the homepage, the user can paste or upload a protein sequence or a set of sequences in FASTA format into the text box. The “BLAST search in” option allows the user to limit the sequence to that from a specific organism. For precise annotation of the query sequence, the “E-value” field allows the user to change the threshold value of the retrieved homologs. As noted above, after the protein sequence has been inputted, CELLO2GO will return four Google-created pie charts: one containing the frequencies of CELLO-predicted localizations and one for each of the three GO annotations, which allows the user to readily visualize the important GO annotation and possible subcellular localizations for the query protein. Taking a multiple sequence set as an example, CELLO2GO returns four pie charts for each ontology (with each associated ontology reported as a percentage) found for the inputted proteins (Figure 1B). The user can check the details by clicking on the number associated with the protein in the table list that appears below the pie charts. When a single sequence is inputted, the output is also displayed as four pie charts but these charts report how often a GO term in an ontology is found in the set of outputted homologs as a percentage (Figure 1C).

## Results and Discussion

**Figure 2:**
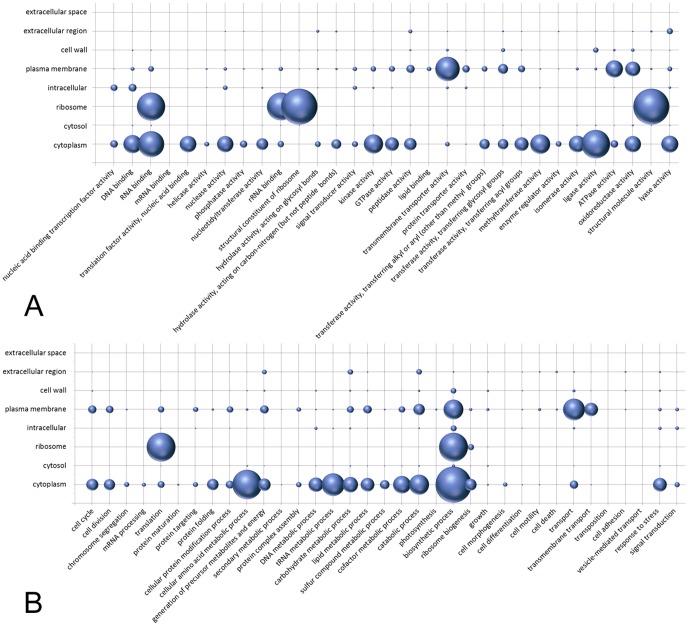
The frequency distributions for the GO slim of the UniProtKB/SwissProt entries in the in-house database. (A) Molecular function (*x* axis) verses cellular component (*y* axis). (B) Biological process (*x* axis) verses cellular component (*y* axis). The size of each sphere is proportional to the number of entries.

We first calculated and present in Figure 2 the statistic distributions for the GO-slim molecular functions (Figure 2A) and biological process (Figure 2B) in relation to their GO cellular components for all bacteria sequences found in the UniProtKB/SwissProt database. Despite the amount of bias in the database, the relationships between the functional annotation and subcellular localizations are clearly seen. For example, proteins with an “RNA binding” as the associated molecular function GO term are usually found in the cytoplasm or are associated with ribosomes. Very few RNA-binding proteins are found associated with the plasma membrane and hardly any are extracellular. Although most proteins function in the cytoplasm (i.e., the GO slim categories, cytoplasm, cytosol, and ribosome), other proteins are found elsewhere, such as those with “transmembrane transporter activities” and “ATPase activities”, which are associated mainly with plasma membranes. Conversely, the relationships for biological processes and subcellular localizations are spread more widely through Fig. 2A than are those of molecular functions and subcellular localizations. When homologous proteins with the same biological process are found by CELLO2GO in the same localization, the results may help determine if the proteins interact or participate in the same pathway. When the protein of interest is found to have a function that is associated with different subcellular localizations, as is the case for certain multifunctional proteins [32], it may be difficult to correlate its correct localizations with its most likely function via examination of the statistical distributions of molecular function/biological processes vs. localization. It is very important to understand a protein functioning from all of its restricted ontology. For example, for the bifunctional protein PuA from Gram negative bacterium *Escherichia coli* (UniProtKB/SwissProt entry P09546) and the multifunctional protein ThiED from Gram positive bacterium *Corynebacterium efficiens* (UniProtKB/SwissProt entry Q8FTH8), CELLO2GO comprehensively and accurately found their GO annotations and made correct subcellular localization prediction.

The overall accuracy for subcellular-localization predictions achieved by CELLO2GO are 99.1% for the Gram-negative bacterial, 99.4% for the Gram-positive bacterial, and 98.4% for the archaeal sequences. Notably, for >50% of the sequences with no GO cellular**-**component annotation, CELLO was able to correctly predict their localizations. Table 1 contains a summary of the GO-annotation coverage correlated with the five subcellular localizations for the Gram-negative bacterial sequences in the PS30GN dataset and the accuracy of CELLO predictions when cellular-component annotations were missing from the BLAST search. The UniProtKB/SwissProt and UniProtKB/TrEMBL databases were separately searched for the three GO annotations for each query. For the PS30GN dataset, which contains well annotated localizations, BLAST easily found annotated homologs for most queries. For the extracellular proteins in the PS30GN dataset, ∼7% could not be associated with a homolog that had a GO cellular-component annotation, whereas for the proteins in the other four localizations, all but <1.5% had homologs with GO cellular component annotations.

**Table 1:** Functional annotation returned by CELLO2GO for Gram-negative bacteria sequence dataset PS30GN.

		Molecular Function		Biological Process		Cellular Component		CELLO^3^
Localization	Number of Proteins	1SwissProt	2TrEMBL	1SwissProt	2TrEMBL	1SwissProt	2TrEMBL	Accuracy
Extracellular	419	32.0	30.5	16.0	14.8	6.7	6.7	53.6
Outer Membrane	541	27.7	27.5	10.4	10.2	1.5	1.3	71.4
Periplasmic	437	13.3	13.3	12.8	12.8	0.5	0.5	50.0
Inner Membrane	1607	22.6	22.5	11.4	11.4	1.4	1.3	52.4
Cytoplasmic	5025	1.4	1.3	1.3	1.3	1.5	1.5	95.9
4PS30GN Overall	8029	9.5	9.4	5.3	5.2	1.5	1.4	77.9

1 The percentage of homologous sequences for which GO functional annotations were not found by a BLAST search of the in-house database derived from the UniProtKB/SwissProt database for bacteria.

2 The percentage of homologous sequences for which GO functional annotations were not found by a BLAST search of the in-house database derived from the UniProtKB/TrEMBL database for bacteria.

3 The percentage of entries for which GO annotations for cellular components were missing or homologs were not retrieved by BLAST searching of the UniProtKB/TrEMBL databases, but for which CELLO accurately predicted the subcellular localization(s).

4 The Gram-negative bacterial benchmark dataset found in PSORTb3.0 [23], denoted PS30GN, includes 8029 protein sequences in five subcellular categories: extracellular, outer membrane, periplasmic, inner membrane, and cytoplasmic.

We also document, in Table 2, the CELLO2GO results for the experimentally derived Gram-negative bacterium, *Pseudomonas aeruginosa* PA01, proteome dataset. At least 30% of the annotations are missing for each ontology. The BLAST search did not find a homologous sequence for one-third of the sequences that could then be used to annotate molecular functions and biological processes of the input proteins. However, CELLO increased the number of localization predictions. The same with PSORTb3.0, we assess the 171 proteins of *Pseudomonas aeruginosa* PA01, which all of them have been ensured in cytoplasmic location experimentally with high confidence [23], and the CELLO prediction alone reaches the prediction recall and precision both 96.5%, which performs almost 5% better than PSORTb3.0 does. Although the number of sequences in the in-house UniProtKB/TrEMBL database is ∼60-fold larger than that in the in-house UniProtKB/SwissProt database, the search of the in-house UniProtKb/TrEMBL database did not annotate many of the sequence not already annotated by the in-house UniProtKB/SwissProt database. Given this observation, the more reliable annotations found in UniProtKB/SwissProt-derived database and the additional computational time required to search the UniProtKB/TrEMBL-derived database, the CELLO2GO default setting searches the UniProtKB/SwissProt-derived database. The CELLO2GO results for the PS30GP and PS30Arch dataset (Table 3 and Table 4, respectively) are presented in the same manner as those for the PS30GN dataset found in Table 1. Similar trends are seen in Tables 1, 2, and 3.

**Table 2:** Functional annotation returned by CELLO2GO for *Pseudomonas aeruginosa* PA01 dataset.

		Molecular Function		Biological Process		Cellular Component		CELLO^3^
Localization	Number of Proteins	1SwissProt	2TrEMBL	1SwissProt	2TrEMBL	1SwissProt	2TrEMBL	Accuracy
Extracellular	94	44.7	41.5	42.6	38.3	30.9	28.7	63.0
Outer Membrane	194	28.9	23.7	20.1	16.5	16.0	12.4	62.5
Outer Membrane Vesicel	338	32.0	28.1	28.1	24.0	27.2	25.1	27.1
Periplasmic	522	24.3	20.7	18.4	15.5	24.7	22.8	51.3
Inner Membrane	1302	38.6	33.5	29.2	24.5	24.7	23.0	82.3
Cytoplasmic	2629	18.6	12.5	19.8	17.2	40.0	39.3	99.1
Unknown Location	1312	68.8	54.2	68.9	61.7	74.9	70.7	-
4 *P.aeruginosa* PA01	5572	35.9	28.3	33.7	29.5	43.1	41.2	-

1 The percentage of homologous sequences for which GO functional annotations were not found by a BLAST search of the in-house database derived from the UniProtKB/SwissProt database for bacteria.

2 The percentage of homologous sequences for which GO functional annotations were not found by a BLAST search of the in-house database derived from the UniProtKB/TrEMBL database for bacteria.

3 The percentage of entries for which GO annotations for cellular components were missing or homologs were not retrieved by BLAST searching of the UniProtKB/TrEMBL databases, but for which CELLO accurately predicted the subcellular localization(s).

4 The proteomic sequence data is that of the newly documented *Pseudomonas aeruginosa* PA01 dataset [31], which contains hypothetical and uncharacterized proteins.

**Table 3:** Functional annotation returned by CELLO2GO for the Gram-positive bacteria dataset PS30GP.

		Molecular Function		Biological Process		Cellular Component		CELLO^3^
Localization	Number of Proteins	1SwissProt	2TrEMBL	1SwissProt	2TrEMBL	1SwissProt	2TrEMBL	Accuracy
Extracellular	312	15.1	15.1	19.2	19.2	2.2	2.2	100.0
Cell wall	82	25.6	22.0	31.7	25.6	9.8	1.2	0.0
Membrane	360	14.7	14.7	4.7	4.7	0.6	0.6	100.0
Cytoplasmic	1822	1.4	1.3	2.0	1.9	2.4	2.4	86.0
4PS30GP Overall	2576	5.7	5.5	5.4	5.2	2.3	2.1	86.8

1 The percentage of homologous sequences for which GO functional annotations were not found by a BLAST search of the in-house database derived from the UniProtKB/SwissProt database for bacteria.

2 The percentage of homologous sequences for which GO functional annotations were not found by a BLAST search of the in-house database derived from the UniProtKB/TrEMBL database for bacteria.

3 The percentage of entries for which GO annotations for cellular components were missing or homologs were not retrieved by BLAST searching of the UniProtKB/TrEMBL databases, but for which CELLO accurately predicted the subcellular localization(s).

4 The Gram-positive bacterial benchmark dataset found in PSORTb3.0 [23], denoted PS30GP, includes 2576 protein sequences in four subcellular categories: extracellular, cell wall, membrane, and cytoplasmic.

**Table 4:** Functional annotation returned by CELLO2GO for archaeal dataset PS30Arch.

		Molecular Function		Biological Process		Cellular Component		CELLO^3^
Localization	Number of Proteins	1SwissProt	2TrEMBL	1SwissProt	2TrEMBL	1SwissProt	2TrEMBL	Accuracy
Extracellular	27	25.9	7.4	74.1	55.6	33.3	33.3	66.7
Cell wall	18	100.0	50.0	100.0	50.0	50.0	44.4	62.5
Membrane	85	27.1	24.7	8.2	7.1	4.7	4.7	75.0
Cytoplasmic	675	0.7	0.4	5.3	5.2	0.9	0.7	100.0
4PS30Arch Overall	805	6.6	4.3	10.1	8.1	3.5	3.2	73.1

1 The percentage of homologous sequences for which GO functional annotations were not found by a BLAST search of the in-house database derived from the UniProtKB/SwissProt database for archaea.

2 The percentage of homologous sequences for which GO functional annotations were not found by a BLAST search of the in-house database derived from the UniProtKB/TrEMBL database for archaea.

3 The percentage of entries for which GO annotations for cellular components were missing or homologs were not retrieved by BLAST searching of the UniProtKB/TrEMBL databases, but for which CELLO accurately predicted the subcellular localization(s).

4 The archaeal benchmark dataset found in PSORTb3.0 [23], denoted PS30Arch, includes 805 protein sequences in four subcellular categories: extracellular, cell wall, membrane, and cytoplasmic.

To show how the CELLO2GO results can be conveniently correlated, we provide Fig. 1B as an example, which displays the results for the 419 PS30GN extracellular proteins that had been submitted. The plus(+)/minus(-) symbols associated with GO term(s) of interest are active and when clicked, the server will respond by showing only those proteins associated with the add/omit GO terms. If the user interest in only proteins with “protein binding” as the Molecular Function annotation and “cell adhesion” as the Biological Process, then 21 sequences, including those for fimbriae and certain secreted serine protease transporters are displayed with their GO terms (Figure 1D). Notably, many of these proteins, e.g., the 354^th^ inputted protein, the serine protease pic autotransporter (GI: 68565646), have been associated with multiple possible subcellular localizations as documented in Q8CWC7 of UniProtKB/SwissProt database. And the CELLO2GO also successfully annotated the localization in outer membrane and extracellular localization when the protein was referred as single localization in original dataset. For most proteins, BLAST in CELLO2GO correctly annotated their cellular component ontology, and CELLO correctly predicted its localization. If the “shift relation” button (top left in Fig. 1D) is clicked, other GO term-related proteins, e.g., flagellum and virulence proteins (from the original list of outputted proteins), are added to the list because either the Molecular Function GO-slim term “protein binding” or the Biological Process GO-slim term “cell adhesion” although not both were assigned to these proteins. By using the “shift relation” button, users can switch between an “either/or” retrieval for “union” as opposed to an “and” retrieval for “intersection”. Sometimes addition of more GO terms can be used to restrict the function or processes of interest, which may eliminate proteins with promiscuous functions. Certain proteins have generally defined GO-slim terms, e.g., those for the 369^th^ inputted protein, bifunctional hemolysin/adenylate cyclase (GI: 34978355). Notably, although hemolysin and cyclase have different functions, both proteins have the GO-slim defined molecular function “ion binding.”

At the same time, the incompleteness and disorderliness of GO based functional annotation for a single protein may occur due to insufficient assay experimentally and too much homologs identified by BLAST, respectively. And both limit the effect of CELLO2GO usage. The later issue could be solved by justified the criteria of E-value strictly.

We also perform CELLO2GO on a dataset derived from a Gram-negative pathogenic bacterium *Vibrio cholerae*. The previous work [33] attempted to identifying some potential drug and vaccine candidates by using complex computational workflow based on comparative and subtractive genomic analysis strategy and pipelining multiple tools. Without carrying out huge computation to confirm unique proteins present in pathogen but absent in host, the CELLO2GO will respond by showing only those proteins associated with the added interest GO terms or omit GO terms with sharing function in host. And some GO terms relative to pathogenic pathway can be further exploited in this case. For example, if a protein was assigned the GO-slim terms “isomerase activity” in Molecular Function ontology, “biosynthetic process”, “cell wall organization or biogenesis” or “cellular amino acid metabolic process” in Biological Process ontology, and “outermembrane” in CELLO prediction simultaneously, then the associated pathogenic pathway may be “D-alanine metabolism”, which involving in bacterial peptidoglycan cell wall synthesis. And the GO-slim terms combination like “transferase activity, transferring alkyl or aryl (other than methyl) group” or “ligase activity” in Molecular Function ontology, “cell wall organization or biogenesis” or “cellular nitrogen compound metabolic process” in Biological Process ontology, and “cytoplasmic” in CELLO prediction may associated with “lipopolysaccharides (LPS) biosynthesis” or “peptidoglycan biosynthesis”, which involving in bacterial endotoxin and host-parasite interaction. The other GO-slim terms “transmembrane transporter activity” in Molecular Function ontology and “cell motility” in Biological Process ontology appearing in one protein may associated with ”bacterial chemotaxis”, which involving in flagellar motor, and the GO-slim term “pathogenesis” may associated with the ability of infection, respectively. The current subcellular localization prediction tools and most existing functional annotation software do not provide any information pipelines on association to specialized proteomic analysis such as potential drug design or biochemical mechanism,

In summary, CELLO2GO can provide brief or detailed annotations of GO categories by combining CELLO localization-prediction and BLAST homology-searching approaches for single or multiple input sequences. When each protein sequence in a query dataset can be confidentially annotated, even though not all proteins in a query set have known localizations, CELLO2GO quickly screens for as many localizations and GO annotations associated with the sequences and collects them as output. CELLO2GO should be a useful tool for research involving complex biological systems.
